# Exploring the relationship between cognition and mental health in intersex participants in the UK Biobank study

**DOI:** 10.1080/13854046.2024.2414478

**Published:** 2024-10-15

**Authors:** Andrew E. Reineberg, Kristen L. Eckstrand, Jason D. Flatt

**Affiliations:** a Institute for Behavioral Genetics, University of Colorado Boulder, Boulder, Colorado, USA; b Department of Radiology, University of Pittsburgh School of Medicine, Magnetic Resonance Research Center, Pittsburgh, Pennsylvania, USA; c Department of Psychiatry, University of Pittsburgh, Pittsburgh, Pennsylvania, USA; d Department of Social and Behavioral Health, University of Nevada, Las Vegas, School of Public Health, Las Vegas, Nevada, USA

**Keywords:** Cognition, mental health intersex, differences in sex development, wellbeing

## Abstract

**Objective:**

The physical and mental health of intersex individuals is woefully understudied. A recent survey of intersex individuals found high rates of self-reported cognitive issues such as difficulty remembering and concentrating as well as high rates of mental health issues, such as depression and anxiety.

**Method:**

The current study explores whether cognitive differences are observed between 353 intersex and over 400,000 non-intersex people using a latent model of cognitive tasks derived from measures in the UK (United Kingdom) Biobank study.

**Results:**

There were no differences in intelligence between intersex people and non-intersex people. We found significantly lower executive function and processing speed in intersex individuals versus non-intersex individuals. However, after accounting for mental health differences *via* regression and case-control matching, there were no significant differences in executive function or processing speed between intersex individuals and non-intersex individuals.

**Conclusion:**

Mental health differences between intersex and non-intersex individuals may account for differences in cognitive factor scores.

## Introduction

### Background on intersex people

Individuals born with differences in sex development (DSD), including those who identify as intersex, are those with differences in sex traits or reproductive anatomy (interACT Statement on Intersex Terminology, n.d.). DSDs include a diverse range of congenital variations relating to differences in gonads/genitals, chromosomes, and endocrine factors (Tamar-Mattis et al., 2018). It is challenging to estimate the prevalence of those living with intersex variations/DSDs because of lack of representation in research and medical care, although estimates are around 2% (Blackless et al., 2000). Intersex/DSD people (labelled as such throughout the rest of the manuscript to reflect the fact that some people with DSDs may not identify as intersex; Committee on Measuring Sex, Gender Identity, & Sexual Orientation, Committee on National Statistics, Division of Behavioral & Social Sciences & Education, & National Academies of Sciences, Engineering, & Medicine, 2022, Ch. 7) face adversity from living with a medical condition and through factors such as shame, stigma, and non-consensual medical and surgical interventions. This adversity has been associated with high rates of mental health conditions in intersex/DSD populations (Zeeman & Aranda, 2020). Although some initiatives have prioritized research on the cognitive and mental health and wellbeing of intersex/DSD people (e.g. the National Institutes of Health Sexual and Gender Minority Research Coordinating Committee), relatively little is known about how those aspects of wellbeing might interact.

### Current research on intersex mental health and cognition

A growing body of evidence suggests intersex/DSD people may be at increased risk for mental health challenges and may experience mental health disparities compared to the general population (reviewed in Zeeman & Aranda, 2020). The dsd-LIFE project, a consortium studying DSDs across 16 European sites, found high rates of unspecified psychiatric disorders and a 3.5 to 6 times increase in suicide attempts when comparing intersex/DSD people to individuals who do not have intersex/DSD traits (Falhammar et al., 2018). These higher rates may be due to shame, stigma, self-esteem, or poor healthcare outcomes (De Vries et al., 2019). U.S. survey data agree that intersex/DSD people experience fair-to-poor general mental health and have high rates of depressive disorder, anxiety disorder, and PTSD diagnoses (Rosenwohl-Mack et al., 2020).

Intersex/DSD people also self-report high rates of cognitive problems such as difficulty concentrating, remembering, or making decisions (Rosenwohl-Mack et al., 2020). Changes in cognition have been associated with certain mental health diagnoses, and preliminary evidence has shown that the cognitive function in intersex/DSD communities may be related to mental health (Rau et al., 2021). Very little additional information is known about the specifics of cognitive differences in intersex/DSD people compared to non-intersex/DSD people.

Cognition can be studied many ways, such as *via* self-report surveys asking about concentration and memory problems, validated self-report questionnaires such as the Behavior Rating Inventory of Executive Function (BRIEF; Gioia et al., 2000), scores on tasks designed to measure specific key components of higher-level cognition (sometimes referred to as executive functions; EFs) such as the ability to inhibit an automatic process or movement (Roberts et al., 1994), and composite scores measuring commonality across many different tasks that rely on EF (e.g. common executive function, a latent construct that captures variability across EF contexts; Miyake et al., 2000). These different types of measurements are not necessarily equivalent (Friedman & Gustavson, 2022; Snyder et al., 2021). Each family of measurements has pros and cons. EFs, as measured by self/parent reports in children, predict future physical health, financial health, and criminal conviction (Moffitt et al., 2011). EFs, as measured by performance on objective tasks, are also important for broad categories of everyday functioning such as success in school, the workplace, relationships, and maintenance of physical health (Diamond, 2013). Batteries of tasks may take a longer time to administer but produce *composite scores or latent variable measures of EF* with good-to-excellent test-retest reliability over as many as six years (Friedman et al., 2016; Gustavson et al., 2018; Suchy & Brothers, 2022). Composites are less susceptible than self-reported measures of cognition to desirability bias. Finally, composite measures of EF maintain strong relevance to everyday functioning, such as a predictor of procrastination (Gustavson et al., 2017) and substance abuse (Gustavson et al., 2017).

The current study utilizes *factor scores* to comprehensively measure latent cognitive ability—including *executive function (EF)* and *intelligence (IQ)*—in an objective (i.e. based on tasks that rely minimally on an individual’s self-evaluation of ability) and reliable manner. We also use a *speed (S)* factor as a measure of simple processing speed for tests of specificity because of the known relationship between processing speed and other measures of cognition (Salthouse, 1996).

Mental health and EF have a complex and intertwined relationship. Performance on task-based measures of EF is impacted by adversity (Miguel et al., 2023) and is affected in many aspects of psychopathology (such as depression, bipolar disorder, PTSD, substance use disorder, and others; Snyder et al., 2015). However, there is a heterogeneous relationship between EF and other aspects of psychopathology (e.g. anxiety; Ibid., Majeed et al., 2023). EF may be a transdiagnostic predictor of psychopathology (i.e. a common process underlying comorbidity among psychiatric disorders; see Hatoum et al., 2023; Snyder et al., 2019 for preliminary evidence). Finally, recent work suggests EF and psychopathology may be bidirectionally linked as evidenced by an EF factor prospectively predicting future internalizing, externalizing, and general psychopathology and vice-versa (Romer & Pizzagalli, 2021). Understanding how cognition varies across multiple domains of measurement, including *latent measures of EF* known for their high reliability and relationship with psychopathology, may help guide future interventions to improve wellbeing across mental and cognitive health (Hack et al., 2023).

Recent calls have been made to improve population health research in diverse communities (Eckstrand et al., 2022). Because intersex/DSD variations often have a biological underpinning, it is easy to attribute differences in cognitive function to the mechanism of the intersex/DSD condition without considering the possibility that cognitive dysfunction may be modifiable. Because cognition may be an important target for managing mental health or vice-versa, understanding the relationships between cognition and mental health in intersex/DSD people is important for exploring strategies to improve wellbeing within intersex/DSD communities.

### Study aims

The current manuscript poses two questions: 1. Do intersex/DSD individuals differ from non-intersex individuals on *latent cognitive abilities*? and 2. Do differences in mental health outcomes account for the differences in cognition between intersex/DSD individuals and non-intersex/DSD individuals?

## Materials and methods

### Participants and procedure

Data for the current study were collected as part of the UK (United Kingdom) Biobank, a large study designed to investigate the determinants of disease in middle and old age (Sudlow et al., 2015). Participants were 353 intersex/DSD individuals and 487910 non-intersex/DSD individuals who completed at least one cognitive assessment. Self-reported ethnicity of the sample was White (90.66%), Mixed (3.72%), Asian or Asian British (3.48%), “Other ethnic group” (0.89%), Black or Black British (0.57%), “Prefer not to answer” (0.32%), Chinese (0.31%), and “Do not know” (0.04%). Cognitive and mental health data from three in-person sessions and an online follow-up session were used. At the assessment visits, participants completed intake, consent, a touch-screen questionnaire, a hearing test, and touchscreen cognitive tests. For the online follow-up, the Fluid Intelligence/Reasoning Test, Pairs Memory Test, and Numeric Memory Test tests were implemented as web-based questionnaires to be completed remotely. The Trail Making Test and Symbol-Digit Substitution Test were added to the online follow-up. See Table 1 for a complete breakdown of when each cognitive task was administered.

### Intersex variable

A categorical intersex variable was created by identifying individuals with intersex related ICD10 codes in UK Biobank field 41202, indicating that an intersex related diagnosis (congenital differences relating to gonads, chromosomes, and/or genitals) was the primary diagnosis recorded from an inpatient medical visit. Intersex-related ICD10 codes were identified from prior work (Kohva et al., 2018). ICD10 codes were used because they are the dominant form of medical records in the UK Biobank sample. The current analysis was unable to determine intersex status in a small subset of Welsh and Scottish data collected between 1981 and 1997, for which only ICD9 codes were available (UK Biobank - Hospital Inpatient Data - Version 3.0, 2018n.d.). The UK Biobank dataset was queried for these ICD10 codes and a binary (0 = non-intersex/DSD, 1 = intersex/DSD) variable was created to represent intersex/DSD status. Intersex/DSD-related ICD10 codes are listed in Supplemental Information. Table S1 contains participant counts for each code.

### Cognitive variables

The current study used the behavioral analysis plan and outcomes from Hatoum et al. (2023; also see Supplementary Information of this reference). Abbreviated methods are provided here. The goal of the behavioral analysis plan was to extract factor scores for executive function (EF), intelligence (IQ), and speed (S) from the UK Biobank’s bespoke battery of “cognitive function tests.” The three-factor approach was chosen as an alternative to creating a single g-factor (as in de la Fuente et al., 2020s analysis of the UK Biobank data) because prior work found genetic relationships between cognitive tasks and outcomes (both psychiatric and educational attainment outcomes) were not fully mediated by the genetic g-factor. This relationship suggests there could be construct-specific (i.e. EF, IQ, S, etc.) associations that are missed by the g-factor (Hatoum et al., 2023). Indeed, the same prior work found, after modelling EF, IQ, and S independently, some outcomes (e.g. educational attainment) had unique genetic relationships with IQ while others (e.g. depression) had a unique genetic relationship with EF.

Many of the UK Biobank’s cognitive function tests are not benchmark neuropsychological executive functions tasks administered in a classical laboratory or clinical setting. However, the tasks included in the EF factor analysis do rely on EF to some extent as evidenced by their correlation amongst one another, with reference measures of EF (e.g. Delis-Kaplan Executive Function System (D-KEFS) Tower Test, Trail Making Test), and strongly, but not perfectly, with a general cognition composite measure in an external validation study conducted in a standard lab setting (Fawns-Ritchie & Deary, 2020). An executive function latent variable may exist outside of a general cognitive factor. Each task has been well-validated within the general population outside the United Kingdom and including those with and without intersex traits and among populations experiencing mental health challenges. Table 1 describes the UK Biobank tasks.

### Prospective memory test

Prior to completing any other cognitive tasks, participants were shown the following instructions: “At the end of the games we will show you four coloured shapes and ask you to touch the Blue Square. However, to test your memory, we want you to actually touch the Orange Circle instead.” The dependent measure was whether participants touched the orange circle on the first try (field 20018). Data from individuals who “abandoned” (field 4287) the test were treated as missing. Fawns-Ritchie and Deary (2020) found that Prospective Memory Test had moderate correlations (based on the Cohen (1992) standard for interpreting correlations) with reference measures of EF and reasoning (D-KEFS Tower Test, COGNITO Matrices test, Trail Making Test B, *r*s = 0.30 − 0.41), justifying our inclusion of data from this task in the EF factor analysis.

### Numeric memory test

Participants were asked to recall numbers of increasing length starting with two digits and ending with up to 12 digits. Duration of presentation for the target numbers was 2s plus 500 ms multiplied by number of digits (e.g. duration for 4-digit number = 2s + 500 ms*4 digits = 4s). Participants were asked to enter the target digits in reverse order using a touchscreen number pad. The dependent measure was the maximum number of digits remembered correctly (field 4282 for in-person and 20240 for online). Data from individuals who “abandoned” the test (coded as a score of −1 and/or “abandoned” in field 4281 for in-person) were treated as missing.

Fawns-Ritchie and Deary (2020) found the Numeric Memory Test had moderate-to-large correlations with a reference digit span task (Wechsler Adult Intelligence Scale-IV Digit Span Backwards subtest, *r* = 0.51), reference Trail Making Test B (*r* = −0.48), and reference measures of EF and reasoning (DKEFS Tower Test (*r* = 0.24) and COGNITO Matrices test (*r* = 0.38)), justifying our inclusion of the data in the EF factor analysis.

### Fluid intelligence/reasoning test

Participants had two minutes to answer as many logic and reasoning questions as possible from a sequence of 13 questions using a touchscreen to respond. For example, the participant might have been shown the sequence “150… 137… 125… 114… 104…” and asked “What comes next?”. Some stimuli were non-numeric (e.g. completion of similes). The dependent measure was the sum of the correct answers (field 20016 for in-person and 20191 for online). Data from individuals who “abandoned” the online version (field 20242) were treated as missing. Fawns-Ritchie and Deary (2020) found the Fluid Intelligence/Reasoning Test had moderate-to-large correlations with a reference digit span task (Wechsler Adult Intelligence Scale-IV Digit Span Forwards subtest (*r* = 0.37), Backwards subtest (*r* = 0.43), Sequencing subtest (*r* = 0.46)) and the COGNITO Matrices test (*r* = 0.38). Data from this task were included in the IQ factor analysis.

### Snap game

The Snap Game is a test of simple processing speed. Participants viewed two cards at a time for two seconds. If the cards matched, participants were instructed to press a button on a button box as quickly as possible. The first five pairs (from a sequence of 12 pairs total) were treated as practice. The dependent measure was the log-transformed mean time to correctly identify matches (field 20023) across the remaining 7 pairs, excluding pairs with RTs < 50 ms (anticipatory responses) and >2,000ms (responses that occurred after cards had disappeared). We excluded mean data from participants who never (*N* = 2449) or always (*N* = 261) pressed the button (based on the maximum and minimum values for number of times the button was pressed in field 403, or 10141 for the pilot phase), as this indicated they were not following instructions. Fawns-Ritchie and Deary (2020) found the Snap Game had a large correlation with a reference measure of simple processing speed (*r* = 0.52). Data from this task were included in the S factor analysis.

### Pairs memory test

In round 1 of this task, participants were given three seconds to memorize matches from a grid of six symbol cards that contained three pairs of matching symbol cards. After three seconds, the cards were turned face down so the symbols were no longer visible. Participants were instructed to touch the screen to point out the matching pairs of symbol cards. Feedback was provided and correct responses (i.e. selecting a matching pair of symbol cards) resulted in the pair of cards disappearing. In round 2, participants were given five seconds to memorize matches from a grid of 12 cards that contained six pairs of matching cards. The response procedure for round 2 was the same as round 1. Possible symbols were: equals sign (=), fir tree (three overlapping triangles), hollow circle, hollow square, rugby-post (H), smiley face, solid circle, heart, diamond, four small squares arranged in a two by two grid, lightning bolt, top hat, linked squares, diagonal cross, crescent, sitting cat, bow tie, house, horse shoe, five-pointed star, person, knife and fork, solid square, triangle, plus sign/cross (+). The dependent measure was the log-transformed number of incorrect matches in the round +1 (field 399 for in-person and 20132 for online), summed across the six- and 12-card rounds. Data were treated as missing if participants did not match all the pairs in a round (e.g. if they abandoned the task; field 398 for in-person and 20131 for online), and the dependent measure was only created for participants who had complete data for both rounds.

This task required participants update working memory as cards were turned over and resist interference from cards viewed across matching attempts and rounds. Fawns-Ritchie and Deary (2020) found the Pairs Memory Test had moderate-to-large correlations with reference measures of EF and reasoning (D-KEFS Tower Test, *r* = −0.40, COGNITO Matrices test, *r* = −0.38, and Trail Making Test B, *r* = 0.28), justifying our inclusion of data from this task in the EF factor analysis.

### Trail Making Test

Participants were instructed to join 25 circles containing numbers 1–25 in ascending order (Trails A) or 25 circles containing number 1–13 and letters A-L in alternating order (1, A, 2, B, …; Trails B) by clicking or touching their chosen response. The dependent measure was the log10-transformed time to complete the alphanumeric set (i.e. Trails B; field 20157) residualized on the log10-transformed time to complete the numeric set (i.e. Trails A; field 20156). Data from this task were included in the EF factor analysis.

### Symbol-digit substitution test

Participants were presented a reference grid containing eight symbols above the digits 1–8. Underneath the reference grid, they were presented a rearranged set of the eight symbols from above. Participants were instructed to select the numbers 1–8 underneath using the reference as a key for correct placement. Responses were made by clicking an on-screen number pad containing only the numbers 1–8. The dependent measure was the number of symbol-digit matches made correctly in one minute (field 20159). Data from individuals who “abandoned” (field 20245) the test were treated as missing.

Prior work has found the task has an executive function component and places demands on working memory, interference control, response selection, and motor control (Cepeda et al., 2013). In a validation study of the UK Biobank tasks using an independent sample, Fawns-Ritchie and Deary (2020) found the Symbol-Digit Substitution Test had a large correlation with the Trail Making Test (Trails B; *r* = 0.56) and moderate-to-large correlation with other reference measures of EF and reasoning (D-KEFS Tower Test and COGNITO Matrices test; *r*s = 0.41) while only having moderate correlation with a reference measure of simple processing speed (*r* = 0.25). Data from this task were included in the EF and S factor analyses.

### Factor analysis

Variables were normally distributed or transformed as noted above in individual task descriptions. Descriptive statistics for each task are provided in Table S1 of Hatoum et al. (2023). After transformation, extreme values were adjusted to +/−4 *SD* from the mean to improve normality while maintaining extreme scores. Variables were rescaled to variance of 1 and reversed when appropriate so high values always corresponded to better performance. Confirmatory factor analysis was conducted in Mplus. We extracted factor scores for EF, IQ and S (described in detail in Supplement 1 of Hatoum et al., 2023). These factors were estimated separately, rather than within the same model, to avoid inflating the correlations among the factor scores. The IQ and S models used full-information maximum likelihood estimation, which uses all available data and assumes missing data are missing at random. For the cEF model, the Prospective Memory Test scores were categorical (pass/fail); we analyzed them with probit link function, which assumes these categories reflect an underlying normal distribution of probability of remembering. These models used means- and variances-adjusted weighted least-squares estimation (WLSMV). For WLSMV, only pairwise deletion is available.

Factor scores were computed for participants with at least one indicator. In the case of individuals who only participated in the first assessment (N_intersex/DSD_ = 162 (45.9%)), their EF factor scores might be based only on their scores for the Pairs Memory Test, as that was the only task given to the full sample. In the case of individuals with multiple assessments including the online battery, their factor scores would be based on a weighted combination of multiple tasks. The latter are better factor scores because more information is available, and the uncorrelated error variances across tasks cancel each other out (their expected mean is zero). Intersex/DSD sample sizes for the individual tasks were as follows: Prospective Memory Test (N = 143), Numeric Memory Test (N = 108), Fluid Intelligence/Reasoning Test (N = 165), Snap Game (N = 353), Pairs Memory Test (N = 353), Trail Making Task (*N* = 74), and Symbol-Digit Substitution Test (*N* = 85). Prior work addressed missingness in the EF factor by comparing and validating the EF factor scores generated from sparsely- versus densely-phenotyped participants and found the scores generated in sparsely-phenotyped participants performed comparably in downstream analyses to scores generated in densely-phenotyped participants (Hatoum et al., 2023).

For the EF model, we were interested in a common factor loading on all tasks (termed common executive functioning in prior work; as in Miyake et al., 2000). Thus, all tasks loaded on the common factor (EF). Four orthogonal task-specific factors were used to account for the fact that some tasks (Prospective Memory Test, Pairs Memory Test, and Numeric Memory Test) were repeated. Orthogonal factors reflect a combination of method variance as well as variance due to processes specific to that paradigm/domain (i.e. uncorrelated with the other tasks in the model). Model fit was good: χ^2^(44) = 1786.53, *p* < .001, CFI = 0.98, RMSEA = 0.01, omega hierarchical (omega_H_) = 0.68. With this sample size (total *N* = 490,588), a large chi-square statistic is expected, but the model fit well by other fit criteria, particularly a CFI > 0.95 and RMSEA < 0.06 (Hu & Bentler, 1998). Omega hierarchical for the EF model was slightly higher than omega hierarchical for a general executive function factor from two reference models (omega_H_ Friedman et al. (2011) = 0.61; omega_H_ Snyder et al. (2021) = 0.57). Model fit was also good for the IQ model (χ^2^(2) = 6.83, *p* = 0.03, CFI = 1.00, RMSEA = 0.003, omega_H_ = 0.88) and the S model (χ^2^(2) = 0.94, *p* = 0.625, CFI = 1.00, RMSEA = 0.000, omega_H_ = 0.78). Factor correlations are as follows: *r*_EF~*S*_ = 0.28 (*p* < 0.001), *r*_EF~IQ_ = 0.45 (*p* < 0.001), *r*_S~IQ_ = 0.21 (*p* < 0.001).

Although it might be tempting to dismiss the EF factor described here as not analogous to the common executive function factor as described in prior works that used more typical batteries of classic EF tasks (e.g. the antisaccade task and Stroop task), preliminary validation work suggested there is overlap with common executive function. In Hatoum et al. (2023), polygenic scores were created for the EF factor in an independent sample of 916 participants from the Colorado Community Twin Sample and Longitudinal Twin Study. Polygenic scores are single values that represent an individual’s cumulative genetic susceptibility for a trait (EF). Polygenic scores are often implemented as the sum of genotypes weighted by effect sizes from genome-wide association studies (Choi et al., 2018). Polygenic score analysis enables cross sample inference because any other dataset with genotype data (e.g. Colorado) can calculate genetic propensity based on the analyses conducted in the initial study (UK Biobank). In this independent sample, polygenic scores for the UK Biobank-derived EF factor predicted reference common executive function scores derived from a battery of nine EF tasks administered at two time points (described in Friedman et al., 2016) controlling for Full Scale IQ, while polygenic scores for the IQ factor predicted Full Scale IQ controlling for EF. This pattern suggests there is overlap between the EF factor described in the current study and a gold standard reference measure of EF and that the EF factor is distinguishable from general cognitive ability.

### Mental health variable

We created a mental health variable relevant to intersex/DSD adults’ experience from the limited data available in the UK Biobank dataset. The creation of the depression variable is documented in Table S1 and Supplementary Information of Border et al., 2019. Briefly, the main outcome of interest was probable lifetime diagnosis of MDD estimated using combined data from the initial in-person touchscreen assessment and an online mental health follow-up assessment. At initial assessment, participants completed a touchscreen implementation of the Structured Clinical Interview for DSM IV Axis I Disorders (First et al., 2002). The derived measure, a binarized version of field 20126 (documented in Smith et al., 2013, the associated UK Biobank documentation is available at https://biobank.ctsu.ox.ac.uk/crystal/crystal/docs/MentalStatesDerivation.pdf, and Border et al., 2019), contrasted individuals with no prior MDD diagnosis (0) to those with either “Probable Recurrent major depression (severe),” “Probable Recurrent major depression (moderate),” or “Probable Single major depression episode” (1). Participants who received a probable lifetime depression diagnosis (1) endorsed a history of at least two weeks of anhedonia or depression accompanied by at least four depression symptoms (symptoms included anhedonia, mood, sleep, fatigue, appetite/weight, feelings of worthlessness, concentration, and ideation) for at-least half the day on almost every day or every day of the at-least two-week period and at least some impairment of study, employment, childcare and housework, or leisure pursuits. The UK Biobank followed up the initial in-person touchscreen assessment of mental health with the *UK Biobank Mental Health Web-Based Questionnaire* (described in Davis et al., 2020) which was modeled after the World Health Organization Composite International Diagnostic Interview (Kessler et al., 1998) to assess history of depression among other aspects of mental health. For online mental health follow-up data, the derived measure used fields 20441, 20446, 20533, 20534, 20533, 20449, 20536, 20450, 20435, 20437, 20439, 20436, and 20440 to create a binarized depression measure equivalent to the measure derived from the initial touch screen assessment as detailed in Border et al. (2019). For regression analyses, we combined the two binary variables to contrast individuals with no data suggesting a probable lifetime MDD diagnosis (0) to those with data from either the initial assessment or mental health follow-up suggesting a probable lifetime MDD diagnosis (1). Table 2 details case counts for the final outcome measure of depression. A total of 162 of 353 intersex individuals completed the mental health assessment at either time point with some completing the mental health assessment at both time points.

### Covariates

Age and a socio-economic status proxy measure were used as covariates in regression analyses. The Townsend Deprivation Index (Townsend et al., 1988) is a measure of material deprivation with higher scores corresponding to higher levels of unemployment and home overcrowding as well as lower levels of car and home ownership in a participant’s postal code.

### Multiple regression

Multiple regression models were run using the Python packages *statsmodels* and *pandas*. To account for running regression models across three cognitive constructs, multiple regression parameters (those presented in Table 3) were considered statistically significant if the *p-*value was less than 0.017 (i.e. 0.05/3).

### Case-control matching

To complement the multiple regression models described above, we performed a follow-up, case-control matched analysis (Breslow & Day, 1980). Case-control matching allows for possible increases in efficiency (i.e. analysis is balanced; Ho et al., 2007). For each intersex/DSD person in the sample, the closest non-intersex/DSD match was selected. A match was defined as a non-intersex/DSD person of the same age, biological sex, and history of probable lifetime MDD diagnosis. The individual with the closest Townsend Deprivation Index score was used. Biological sex was derived from a combination of medical records and patient self-reporting. The UK Biobank biological sex variable (field 31) contains only female/male options (no intersex option). Prior work has shown a mismatch between biological sex from UK Biobank field 31 and chromosomal sex in a subset of intersex/DSD UK Biobank participants (Ackley et al., 2023). General weaknesses of the case-control matching design are the rendering of all unmatched control data useless and possible creation of a non-intersex/DSD subsample that is not representative of the population (Rose & van der Laan, 2009).

## Results

Table 2 contains demographic details of the intersex/DSD and non-intersex/DSD sample. The intersex/DSD subsample was younger than non-intersex/DSD sample (*t*(1, 488261) = −6.65, *p* < 0.001). Intersex/DSD participants had higher material deprivation (*t*(1, 487662) = 2.46, *p* = 0.014) but equivalent income with non-intersex/DSD participants *t*(1, 487662) = 1.18, *p* = 0.238). Intersex/DSD individuals were more likely to have a probable lifetime MDD diagnosis (*t*(1, 219750) = 3.32, *p* = 0.001) than non-intersex/DSD individuals. Without controlling for age and/or material deprivation, there were no significant differences between intersex/DSD individuals and non-intersex/DSD individuals in EF, IQ, or S factor scores.

Table 3 contains the results of multiple regression analyses. We conducted five multiple regression analyses for each of three cognitive factors. In model 1, we ran a naïve model regressing cognition on intersex/DSD status, age, and Townsend Deprivation Index. In models 2–4, we regressed cognition on intersex/DSD status, age, and Townsend Deprivation Index as well as confounding cognitive factors or probable lifetime MDD diagnosis in separate models to diagnosis the effects of each confounding factor or mental health separately. In model 5, we ran a full model by regressing cognition on intersex/DSD status, age, Townsend Deprivation Index, confounding cognitive factors, and probable lifetime MDD diagnosis.

Intersex/DSD status was not significantly associated with IQ factor scores in any regression model (Table 3f–j).

Model 1 results (analyses controlling for age and Townsend Deprivation Index): After accounting for only age and Townsend Deprivation Index, intersex individuals had lower EF factor scores (*t*(3, 476445) = −3.02, *p* = 0.003) and lower S factor scores (*t*(3, 482521) = −2.46, *p* = 0.014) than non-intersex individuals (Table 3a,k).

Model 2–4 results (models with a single additional confound of interest): Intersex/DSD status was still associated with lower EF factor scores after adding S factor scores (Table 3b; t(4, 474101) = −2.58, *p* = 0.010) and IQ factor scores (Table 3c; t(4, 243731) = −2.54, *p* = 0.011) but was not associated with lower EF factor scores after adding probable lifetime MDD diagnosis (Table 3d; t(4, 218190) = −2.28, *p* = 0.023) as covariates in separate models. Intersex/DSD status was not associated with lower S factor scores after adding EF factor scores (Table 3l; t(4, 474101) = −1.83, *p* = 0.068) and IQ factor scores (Table 3m; t(4, 243010) = −2.21, *p* = 0.027), or probable lifetime MDD diagnosis (Table 3n; t(4, 218030) = −1.81, *p* = 0.071) as covariates in separate models.

Model 5 results for EF (full models with all covariates): For the full model in which age, Townsend Deprivation Index, S factor scores, and IQ factor scores were controlled for, there were no significant differences in EF factor scores between intersex/DSD and non-intersex/DSD individuals (Table 3e; t(6, 182548) = −2.11, *p* = 0.035). A one degree-of-freedom *F*-test confirmed the full model including probable lifetime MDD diagnosis fit better than a model with only age, Townsend Deprivation Index, S factor scores, and IQ factor scores (*F*(1, 182541) = 54.97, *p* < 0.001).

Model 5 results for S (full models with all covariates): For the full model in which age, Townsend Deprivation Index, EF factor scores, and IQ factor scores were controlled for, there were no significant differences in S factor scores between intersex/DSD individuals (Table 3o; t(6, 182548) = −0.82, *p* = 0.414). A one degree-of-freedom *F*-test confirmed the full model including probable lifetime MDD diagnosis fit better than a model with only age, Townsend Deprivation Index, EF factor scores, and IQ factor scores (*F*(1, 182541) = 76.68, *p* < 0.001).

To follow up the multiple regression models in Table 3, we performed a case-control analysis to see if there were any intersex/DSD differences in cognition after matching the intersex/DSD sample with the non-intersex/DSD sample on age, biological sex, Townsend Deprivation Index, and probable lifetime MDD diagnosis. After matching, there were no significant intersex/DSD versus non-intersex/DSD differences in EF factor scores (*t*(1, 321) = −1.566, *p* = 0.118), IQ factor scores (*t*(1, 321) = −1.440, *p* = 0.151), or S factor scores (*t*(1, 321) = −1.182, *p* = 0.238).

## Discussion

### General discussion

The current study examined the cognitive functioning of intersex/DSD people using a battery of tasks and a three-factor model of cognition (EF, IQ, and S). We found more probable lifetime MDD diagnoses among intersex/DSD people. Intersex/DSD status was not associated with differences in IQ factor scores in regression models. In regression models that did not account for mental health disparities but accounted for age and material deprivation as a proxy for socioeconomic status, intersex/DSD people had slightly lower EF and S factor scores than non-intersex/DSD participants. Differences in EF factor scores between intersex/DSD and non-intersex/DSD people persisted even after controlling for IQ or S factor scores. Differences in S factor scores between intersex/DSD and non-intersex/DSD individuals did not persist after controlling for EF or IQ factor scores. These results corroborate prior survey results in which more than half (56.6%) of an intersex/DSD sample endorsed “serious difficulty concentrating, remembering, or making decisions” (Rosenwohl-Mack et al., 2020).

*After accounting for mental health via multiple regression and case-control matching, the differences in EF and S factor scores between intersex/DSD individuals and non-intersex/DSD individuals were not significant*. Mental health was a significant predictor of cognitive performance (EF, IQ, and S) in every model it was included in. Single degree-of-freedom model comparisons showed the inclusion of mental health significantly improved model fit when predicting EF and S factor scores (not tested for IQ factor scores due to lack of significant intersex/DSD effects in this cognitive domain).

Our results provide a new level of specificity regarding cognitive health in intersex/DSD communities. First, in our study, intersex/DSD people only had differences in EF and S factor scores, but not in IQ factor scores. Second, differences in S factor scores did not persist after accounting for EF and IQ. However, there were still differences in EF factor scores after accounting for S and IQ. Third, differences in EF and S factor scores were accounted for by mental health measures, suggesting cognitive differences in intersex/DSD individuals may be explained by mental health challenges.

Our study highlights the need for increased access to mental health support services for the intersex community because after controlling for mental health, there were no significant differences in EF and S factor scores. Research has shown that interventions such as mindfulness meditation (Banks et al., 2015; Dunning et al., 2018; Jones et al., 2019) and physical activity (Lubans et al., 2016) can improve mental health and cognitive functioning. While many cognitive and mental health interventions have been primarily studied in communities who do not have known intersex/DSD traits, interventions such as these could prove fruitful in the intersex/DSD community.

Another potentially fruitful area of intervention is metacognitive awareness. Intersex/DSD people self-report difficulty concentrating, remembering, and making decisions (Rosenwohl-Mack et al., 2020). These self-report measures reflect individual’s perceptions of their own ability. The current study found only small differences in EF and S factor score in naïve models and no significant differences after accounting for MDD. To address this discrepancy between perceived and measured cognitive ability, intersex/DSD people experiencing mental health challenges may benefit from interventions targeting one component of metacognition, knowledge of cognition (Schraw, 1998), which is a modifiable skill encompassing knowledge of factors that affect learning, useful strategies for learning and performing, and when/why to use cognitive skills.

Neuropsychologists should consider intersex/DSD status when developing an assessment plan or neuropsychological intervention. Several past studies have identified best practices in how to ask about intersex traits, such as asking the following question: “Have you ever been diagnosed by a medical doctor or other health professional with an intersex condition or a “Difference of Sex Development (DSD)” or were you born with (or developed naturally in puberty) genitals, reproductive organs, and/or chromosomal patterns that do not fit standard definitions of male or female?” (Committee on Measuring Sex, Gender Identity, & Sexual Orientation, Committee on National Statistics, Division of Behavioral & Social Sciences & Education, & National Academies of Sciences, Engineering, & Medicine, 2022; Flatt & Cicero, 2023; Tamar-Mattis et al., 2018). Options for answering should include “yes/no/don’t know” and “prefer not to answer”.

It is important that generalization of the present results be limited to individuals with intersex/DSD traits. While all individuals who have intersex/DSD traits also have a gender identity—a person’s deeply felt and intrinsic sense of their own gender—sex and gender identity are distinct facets of human experience. The present results should not be generalized to populations with gender identity diversity, although future work could explore the relationship between EF and mental health in other sexual and gender minority groups (NIH Fy 2016–2020 Strategic Plan to Advance Research on the Health and Well-Being of Sexual and Gender Minorities).

### Limitations

A major limitation of the current study is use of intersex/DSD-related primary diagnosis from an inpatient medical visit to identify intersex/DSD people. The current results may or may not generalize to intersex individuals who are not aware of their own intersex/DSD variation, have not sought medical treatment related to an intersex/DSD variation, or did not receive an ICD10-based diagnosis related to an intersex/DSD variation. Another limitation of the current study is the inability to capture intersex/DSD status based on medical records prior to UK Biobank data collection (e.g. intersex/DSD individuals recruited after they had medical care related to their intersex/DSD variation).

In the current study, intersex is an umbrella term of 58 unique medical diagnosis codes. Intersex/DSD people deserve high-quality research that recognizes this heterogeneity and focuses on improving their health. Mental health care and interventions may need to be personalized rather than to intersex/DSD persons collectively. Recognizing heterogeneity within other diagnosis families (e.g. subtypes of depression) has yielded valuable insights allowing for treatment customizations.

We only considered latent measures of executive function, intelligence, and speed. Future work could measure other facets of cognition, such as memory and language. Additionally, cognitive challenges in the intersex/DSD community may be task- or ability-specific, which should be explored with future task and item-based analyses in the UK Biobank and in samples recruited specifically to explore heterogeneity in cognitive impairment. Finally, change and potential cognitive decline should be considered among intersex populations in longitudinal studies.

Another major limitation of utilizing data from consortia, such as the UK Biobank, is depth of phenotyping. Although validation work suggests the EF factor used in the current study is related to a common executive function latent factor (Hatoum et al., 2023), an obvious criticism of this work is that the UK Biobank was not designed to measure common executive function with standard EF tasks, such as the antisaccade and Stroop tasks (as in Friedman et al., 2016; Miyake et al., 2000). Additionally, the UK Biobank cognitive task battery places broad demands on cognition that could be modelled in many ways with alternative statistical approaches. A related major limitation is high missingness, particularly regarding cognitive tasks that were used in the EF factor model. Approximately 45% of the intersex sample’s EF factor scores were based on a single test, the Pairs Matching Test. Missingness in the UK Biobank is driven by many factors. Some tasks are missing for large blocks of participants because they were added part of the way through data collection. Data may be missing at random (i.e. missingness is related to observed data). For example, participants without access to transportation or those experiencing mental health troubles may not be able to or may not want to participate in follow-up visits. Limited prior work in the UK Biobank dataset suggests a pattern of missing at random is present within the UK Biobank dataset (Campbell & Cullen, 2023) and motivated inclusion of age, Townsend Deprivation Index, and MDD in regression models in the current study. Two prior studies showed minimal difference between performance of UK Biobank phenotypes from complete versus incomplete datasets in downstream analyses (Cullen et al., 2015; Hatoum et al., 2023). It is still possible unobserved factors or factors not included in regression models are related to missingness (i.e. missing not at random). In this case, parameter estimates will be biased until all mechanisms of missing data are modeled. Given the many known issues that can arise due to missingness (e.g. biased parameter estimates, impacted generalizability), the current study should be replicated in future analyses of the UK Biobank sample as more cognitive data become available.

A statistical limitation of the current study is mismatch in sample sizes across multiple regression models. The effect of intersex status on EF and S in the models that included depression could be due to the reduced power from smaller sample sizes rather than the addition of the mental health variables of interest as interpreted above. Finally, the effect of intersex/DSD status on EF fell below the threshold of statistical significance established for this study and the effect size was small (β = −0.10) yet it may still be considered a trend of interest.

Considering these limitations, our results should be interpreted with caution until the weaknesses noted above can be addressed by a replication study. Future research in this area should strive to improve in three ways. First, sampling should be improved. There should be an effort to collect well-powered samples of intersex/DSD people within diagnosis categories to understand mental and cognitive health more accurately within homogeneous groups. Targeted recruitment of intersex/DSD individuals may be necessary rather than relying on available datasets in which intersex/DSD participants can only be identified if they have a history of seeking medical care related directly to intersex/DSD variations. Future studies using the UK Biobank dataset should include individuals with an intersex/DSD-related secondary diagnosis from an inpatient (or outpatient) medical visit (e.g. by analyzing UK Biobank field 41204). Second, quality of phenotyping should be addressed. EF and mental health outcomes should be based on reference measures or, ideally, batteries of reference measures if latent variables are of interest. Assessments should be administered in a standard laboratory or clinical context, and effort should be dedicated to address missingness (i.e. ensure all participants complete as many tasks as possible if a battery-based approach is used). Future work should consider aspects of mental health beyond MDD and aspects of cognition beyond the three cognitive constructs used in the current study. Third, additional statistical approaches should be considered. Future work should explore the possibility that mental health formally mediates the relationship between intersex/DSD status and neuropsychological function.

## Conclusions

Our results align with prior work showing mental health disparities and differences in cognition between intersex/DSD and non-intersex/DSD people. Mental health disparities may be affecting cognitive health. Intersex/DSD and non-intersex/DSD individuals did not differ in IQ factor scores but did differ in EF and S factor scores. After accounting for MDD, there were no significant differences between intersex/DSD individuals and non-intersex/DSD individuals. Poor mental health (i.e. MDD)—and not the underlying cause of intersex/DSD variation - may be a source of cognitive concerns. There is a broad need to promote awareness about these disparities. Additional research in this area may support the pursuit of intersex/DSD tailored neuropsychological interventions.

## Supplementary Material

Supplement

## Figures and Tables

**Table 1.** Cognitive task information.

Task	Task Type	Test-retest Reliability Pearson’s r from Fawns-Ritchie and Deary (2020)	Included in model of:	Administered
Trail Making Test (A/B)	executive function	0.478/0.775	EF	O
Symbol-Digit Substitution Test	complex processing speed	0.583	EF, S	O
Prospective Memory Test	prospective memory	0.453	EF	IN, R, IM
Pairs Memory Test	visual memory, working memory	0.409	EF	IN, R, IM, O
Numeric Memory Test	working memory	0.501	EF	IN, IM, O
Fluid Intelligence/Reasoning Test	reasoning and logic	0.445	IQ	IN, R, IM, O
Snap Game	simple processing speed	0.55	S	IN, R, IM

Notes: Test-retest reliability is Pearson’s r from Fawns-ritchie and Deary (2020). EF = executive function, S = speed, IQ = intelligence, O = online follow-up session, IN = initial in-person visit, R = repeat follow-up visit, IM = imaging visit. Descriptive statistics are available in Table S1 of Hatoum et al. (2023).

**Table 2.** Demographics.

	Intersex/DSD	Non-intersex/DSD
Sample Size	353	487910
Mean Age (SD)*	53.67 (8.54)	56.54 (8.09)
Biological Sex Counts	170 Males, 183 Females	223389 Males, 264521 Females
Mean Townsend Deprivation Index (SD)*	−0.90 (3.28)	−1.31 (3.09)
Mean Income	2.02 (1.91)	1.89 (2.10)
Probable Lifetime Depression Diagnosis*	64 probable diagnosis,98 no diagnosis; 39.5%	61073 probable diagnosis, 158517 no diagnosis; 27.81%
Mean Intelligence Factor Score (SD)	−0.09 (0.80)	0.00 (0.82)
Mean Executive Function Factor Score (SD)	−0.07 (0.61)	−0.03 (0.57)
Mean Speed Factor Score (SD)	−0.01 (0.83)	0.00 (0.75)

Notes: DSD = differences in sex development, SD = standard deviation. Townsend Deprivation Index range = −6.26 (least deprived) to 11.00 (most deprived). Income range = −3 (lowest income) to 5 (highest income). Executive Function factor score range = −3.85 to 3.16. Intelligence factor score range = −2.79 to 3.42. Speed factor score range = −3.77 to 2.55. Note: Biological sex noted here is from UK Biobank field 31, which contains only female/male options (no intersex option) derived from a combination of medical records and patient self-reporting.

* denotes significant group differences as described in main text.

**Table 3.** Regression results.

			Intersex/DSD		Age		Townsend Deprivation Index		Speed Factor Score		Intelligence Factor score		Probable Lifetime MDD Diagnosis	
	DV	n	β	t (p)	β	t (p)	β	t (p)	β	t (p)	β	t (p)	β	t (p)
a	EF	476445	−0.09	−3.02 (0.003)	−0.02	−191.20 (<0.001)	−0.02	−71.14 (<0.001)						
b	EF	474101	−0.07	−2.58 (0.010)	−0.01	−136.78 (<0.001)	−0.01	−54.33 (<0.001)	0.16	144.08 (<0.001)				
c	EF	243731	−0.10	−2.54 (0.011)	−0.02	−160.09 (<0.001)	−0.01	−32.47 (<0.001)			0.34	238.87 (<0.001)		
d	EF	218190	−0.11	−2.28 (0.023)	−0.02	−150.19 (<0.001)	−0.02	−47.92 (<0.001)					−0.01	−4.82 (<0.001)
e	EF	182548	−0.10	−2.11 (0.035)	−0.02	−106.99 (<0.001)	−0.01	−21.05 (<0.001)	0.15	82.41 (<0.001)	0.31	191.05 (<0.001)	−0.02	−7.41 (<0.001)
			Intersex/DSD		Age		Townsend Deprivation Index		Speed Factor Score		Intelligence Factor score		Probable Lifetime MDD Diagnosis	
		n	β	t (p)	β	t (p)	β	t (p)	β	t (p)	β	t (p)	β	t (p)
f	IQ	243796	−0.10	−1.67 (0.096)	−0.01	−47.22 (<0.001)	−0.04	−63.19 (<0.001)						
g	IQ	243010	−0.07	−1.21 (0.228)	−0.003	−11.89 (<0.001)	−0.03	−53.50 (<0.001)	0.21	94.89 (<0.001)				
h	IQ	243731	−0.02	−0.40 (0.691)	0.01	26.13 (<0.001)	−0.02	−42.25 (<0.001)			0.56	238.87 (<0.001)		
i	IQ	183119	−0.08	−1.14 (0.255)	−0.01	−41.55 (<0.001)	−0.03	−48.89 (<0.001)					0.03	7.80 (<0.001)
j	IQ	182548	0.01	0.10 (0.924)	0.01	32.50 (<0.001)	−0.02	−29.03 (<0.001)	0.09	37.49 (<0.001)	0.54	191.05 (<0.001)	0.04	11.50 (<0.001)
			Intersex/DSD		Age		Townsend Deprivation Index		Speed Factor Score		Intelligence Factor score		Probable Lifetime MDD Diagnosis	
		n	β	t (p)	β	t (p)	β	t (p)	β	t (p)	β	t (p)	β	t (p)
k	S	482521	−0.09	−2.46 (0.014)	−0.03	−242.10 (<0.001)	−0.03	−75.68 (<0.001)						
l	S	474101	−0.07	−1.83 (0.068)	−0.03	−199.69 (<0.001)	−0.02	−61.44 (<0.001)	0.27	144.08 (0.001)				
m	S	243010	−0.11	−2.21 (0.027)	−0.03	−177.82 (<0.001)	−0.02	−36.26 (<0.001)			0.16	94.89 (<0.001)		
n	S	218030	−0.10	−1.81 (0.071)	−0.03	−172.10 (<0.001)	−0.02	−40.04 (<0.001)					−0.03	−10.13 (<0.001)
o	S	182548	−0.05	−0.82 (0.414)	−0.03	−125.08 (<0.001)	−0.01	−24.23 (<0.001)	0.24	82.41 (<0.001)	0.08	37.49 (<0.001)	−0.03	−8.76 (<0.001)

Notes: Results are organized as one model per row (a-l). DV = Dependent Variable, N = sample size, DSD = differences in sex development. Boldface = p < 0.017 (0.05/3; a Bonferroni correction for running one group of multiple regression models for each of three cognitive factors).
