# Supplementary material for: Exploring the relationship between cognition and mental health in intersex participants in the UK Biobank study
Source: Clin Neuropsychol. Author manuscript; Available in PMC 2026 Apr 1. (PMC11997161; doi:10.1080/13854046.2024.2414478)
Supplement: Supplement [file NIHMS2051234-supplement-Supplement.docx]

**Supplemental Information**

The UK Biobank was queried using the following intersex/DSD-related ICD10 codes to create a binary (0 = non-intersex/DSD, 1 = intersex/DSD) variable: Q50, Q500, Q5001, Q5002, Q501, Q502, Q503, Q5031, Q5032, Q5039, Q504, Q505, Q506, Q51, Q510, Q511, Q5110, Q5111, Q512, Q5121, Q5122, Q5128, Q513, Q514, Q515, Q516, Q517, Q518, Q5181, Q51810, Q51811, Q51818, Q5182, Q51821, Q51828, Q519, Q52, Q520, Q521, Q5210, Q5211, Q5212, Q52120, Q52121, Q52122, Q52123, Q52124, Q52129, Q522, Q523, Q524, Q525, Q526, Q527, Q5270, Q5271, Q5279, Q528, Q529, Q53, Q530, Q5300, Q5301, Q5302, Q531, Q5310, Q5311, Q53111, Q53112, Q5312, Q5313, Q532, Q5320, Q5321, Q53211, Q53212, Q5322, Q5323, Q539, Q54, Q540, Q541, Q542, Q543, Q544, Q548, Q549, Q55, Q550, Q551, Q552, Q5520, Q5521, Q5522, Q5523, Q5529, Q553, Q554, Q555, Q556, Q5561, Q5562, Q5563, Q5564, Q5569, Q557, Q558, Q559, Q56, Q560, Q561, Q562, Q563, Q564, Q64, Q640, Q641, Q6410, Q6411, Q6412, Q6419, Q642, Q643, Q6431, Q6432, Q6433, Q6439, Q644, Q645, Q646, Q647, Q6470, Q6471, Q6472, Q6473, Q6474, Q6475, Q6479, Q648, Q649, E345, E25, E250, E2500, E2501, E259, Q96, Q960, Q961, Q962, Q963, Q964, Q965, Q966, Q967, Q968, Q969, Q970, Q971, Q972, Q973, Q974, Q975, Q976, Q977, Q978, Q979, Q980, Q98, Q981, Q982, Q984, Q985, Q986, Q987, Q988, and Q989.

**Table S1. Participant ICD10 code counts^[[1]](#footnote-1)^**

| **ICD10 code** | **Diagnosis** | **N** |
| --- | --- | --- |
| **Q50** | Congenital malformations of ovaries, fallopian tubes and broad ligaments | 86 |
| **Q501** | Developmental ovarian cyst | 1 |
| **Q503** | Other congenital malformations of ovary | 1 |
| **Q504** | Embryonic cyst of fallopian tube | 70 |
| **Q505** | Embryonic cyst of broad ligament | 13 |
| **Q506** | Other congenital malformations of fallopian tube and broad ligament | 1 |
| **Q51** | Congenital malformations of uterus and cervix | 31 |
| **Q511** | Doubling of uterus with doubling of cervix and vagina | 1 |
| **Q512** | Other doubling of uterus | 12 |
| **Q513** | Bicornate uterus | 6 |
| **Q514** | Unicornate uterus | 1 |
| **Q518** | Other congenital malformations of uterus and cervix | 10 |
| **Q519** | Congenital malformation of uterus and cervix, unspecified | 2 |
| **Q52** | Other congenital malformations of female genitalia | 44 |
| **Q520** | Congenital absence of vagina | 2 |
| **Q521** | Doubling of vagina | 10 |
| **Q522** | Congenital rectovaginal fistula | 1 |
| **Q524** | Other congenital malformations of vagina | 4 |
| **Q525** | Fusion of labia | 19 |
| **Q526** | Congenital malformation of clitoris | 1 |
| **Q527** | Other and unspecified congenital malformations of vulva | 4 |
| **Q528** | Other specified congenital malformations of female genitalia | 4 |
| **Q53** | Undescended and ectopic testicle | 65 |
| **Q530** | Ectopic testis | 5 |
| **Q531** | Undescended testicle, unilateral | 41 |
| **Q532** | Undescended testicle, bilateral | 9 |
| **Q539** | Undescended testicle, unspecified | 11 |
| **Q54** | Hypospadias | 37 |
| **Q540** | Hypospadias, balanic | 11 |
| **Q541** | Hypospadias, penile | 6 |
| **Q544** | Congenital chordee | 1 |
| **Q548** | Other hypospadias | 1 |
| **Q549** | Hypospadias, unspecified | 22 |
| **Q55** | Other congenital malformations of male genital organs | 40 |
| **Q550** | Absence and aplasia of testis | 2 |
| **Q551** | Hypoplasia of testis and scrotum | 1 |
| **Q552** | Other and unspecified congenital malformations of testis and scrotum | 13 |
| **Q554** | Other congenital malformations of vas deferens, epididymis, seminal vesicles and prostate | 7 |
| **Q555** | Congenital absence and aplasia of penis | 1 |
| **Q556** | Other congenital malformations of penis | 13 |
| **Q558** | Other specified congenital malformations of male genital organs | 3 |
| **Q64** | Other congenital malformations of urinary system | 40 |
| **Q641** | Exstrophy of urinary bladder | 1 |
| **Q642** | Congenital posterior urethral valves | 1 |
| **Q643** | Other atresia and stenosis of urethra and bladder neck | 24 |
| **Q644** | Malformation of urachus | 10 |
| **Q646** | Congenital diverticulum of bladder | 1 |
| **Q647** | Other and unspecified congenital malformations of bladder and urethra | 4 |
| **E345** | Androgen insensitivity syndrome | 2 |
| **E25** | Congenital adrenogenital disorders associated with enzyme deficiency | 11 |
| **E250** | Congenital adrenogenital disorders associated with enzyme deficiency | 9 |
| **E259** | Adrenogenital disorder, unspecified | 1 |
| **Q96** | Turner's syndrome | 3 |
| **Q969** | Turner's syndrome, unspecified | 3 |
| **Q980** | Klinefelter syndrome karyotype 47, XXY | 2 |
| **Q98** | Other sex chromosome abnormalities, male phenotype, not elsewhere classified | 11 |
| **Q984** | Klinefelter syndrome, unspecified | 9 |
| **Q988** | Other specified sex chromosome abnormalities, male phenotype | 1 |
|  | total unique participants | 353 |

1. ICD10 = International Classification of Diseases (10^th^ revision), N = sample size [↑](#footnote-ref-1)
